# Pituitary Apoplexy Following Elective Total Hip Replacement

**DOI:** 10.7759/cureus.40600

**Published:** 2023-06-18

**Authors:** Aspin A Makadia, William Jenkins, Farhan Azad, Remon Bebawee

**Affiliations:** 1 Internal Medicine, University at Buffalo, Buffalo, USA

**Keywords:** transsphenoidal resection, total hip arthroplasty, nonfunctioning pituitary adenoma, pituitary macroadenoma, pituitary apoplexy

## Abstract

Pituitary apoplexy is an endocrine-related emergency most commonly caused by hemorrhage into a preexisting pituitary adenoma. Known risk factors for such hemorrhage include major surgical procedures, head trauma, pregnancy, anticoagulation, and the administration of hormone therapies for the correction of primary hypothalamic deficiencies. Elective orthopedic surgery is an uncommon precipitator of pituitary apoplexy that is rarely described. We report the case of a patient with a previously unknown pituitary macroadenoma who developed apoplexy as a complication of elective right total hip arthroplasty.

## Introduction

Pituitary apoplexy (PA) is an endocrinologic emergency typically arising from hemorrhage into a preexisting pituitary adenoma, although it may occur in a non-adenomatous pituitary. Known precipitating factors include major surgery (typically with significant hemodynamic shifts), pregnancy, anticoagulation, head injury, stereotactic irradiation, coagulopathy secondary to liver failure, and administration of thyrotropin-releasing hormone (TRH), gonadotropin-releasing hormone (GnRH), and dopamine agonists [1,2]. The development of PA is associated with nonfunctioning macroadenomas, which predispose to symptomatic or clinical PA. The incidence of PA in patients with pituitary macroadenoma is between 2% and 7%, whereas the incidence of asymptomatic or subclinical PA is around 25% [3]. PA can occur from the first to the ninth decade of life, with a peak incidence during the fifth decade. There is no sex predominance or histological subtype of pituitary tumor that carries a higher risk of PA [4]. There have only been a few case reports of PA following elective surgery without large hemodynamic shifts or intraoperative complications. We report the case of a 52-year-old man who developed postoperative PA after an uncomplicated right total hip arthroplasty requiring emergent transsphenoidal resection and endocrine hormone replacement.

## Case presentation

**Figure 1:**
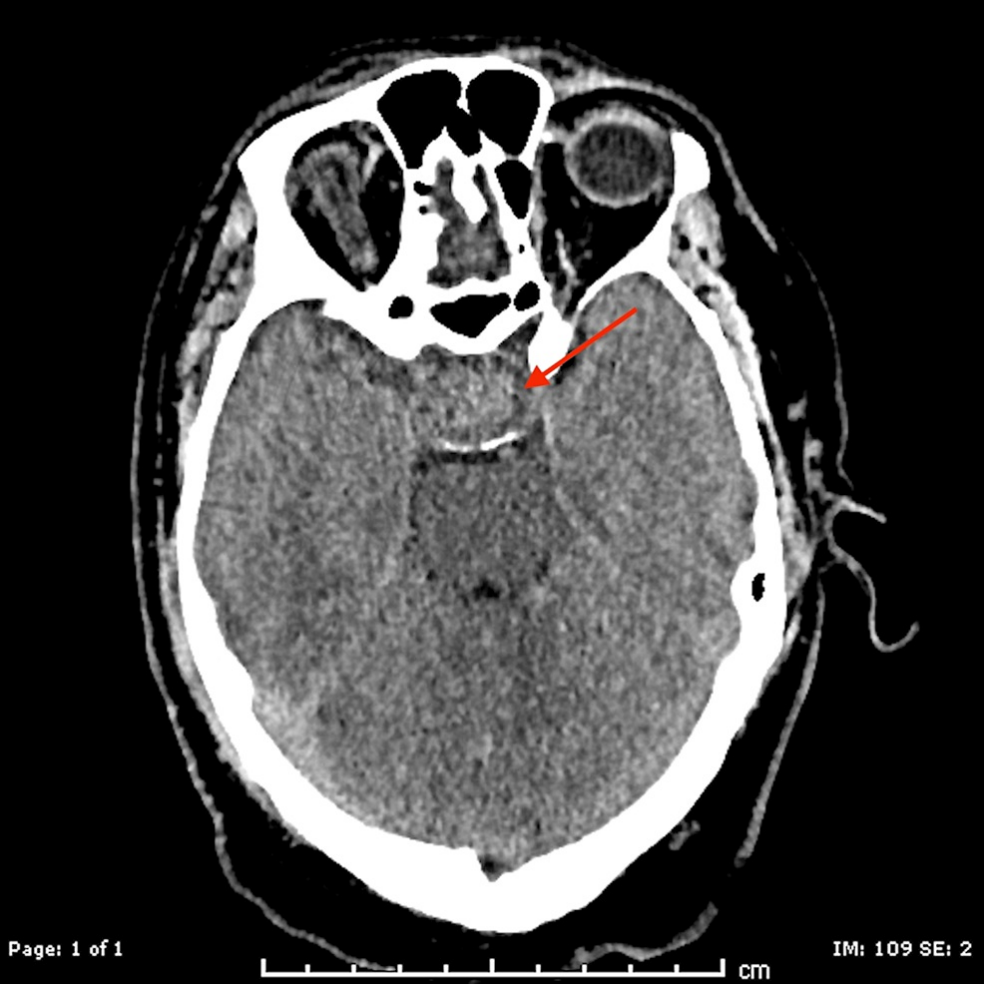
CT head taken after the initial development of new neurological findings on physical exam. The arrow highlights a heterogeneous soft tissue mass displaying characteristics consistent with pituitary macroadenoma. CT: computed tomography

A 52-year-old Caucasian male with a history of hypothyroidism, hypogonadism, hypertension, and prior left hip replacement presented for elective total right hip replacement. The procedure was performed with a posterior approach, with minimal estimated blood loss and no intraoperative complications. On postoperative day two, it was noticed that the patient was having episodes of intermittent nausea and vomiting, later accompanied by frontal headaches, diplopia, photophobia, and phonophobia. At that time, a physical exam elucidated a new right eye ptosis and fixed gaze palsy. A computed tomography (CT) stroke study was performed, which showed an enlarged soft tissue heterogeneous mass with a moderately expanded sella turcica (Figure 1). 

**Figure 2:**
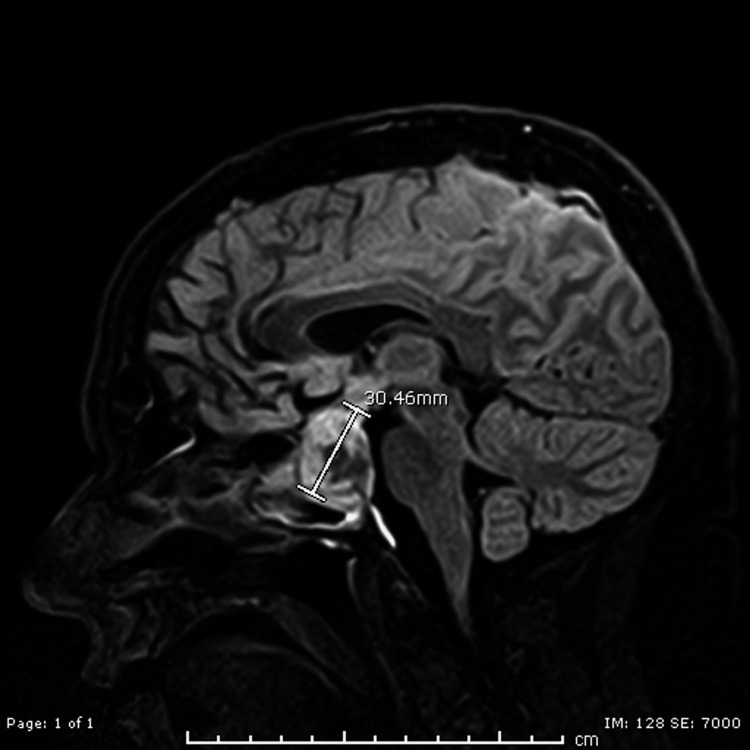
Sagittal cut of MRI brain DWI-FLAIR, which shows pituitary macroadenoma that measures 30.46 millimeters at its longest angle. MRI: magnetic resonance imaging; DWI-FLAIR: diffusion-weighted imaging and fluid-attenuated inversion recovery

Subsequent magnetic resonance imaging (MRI) of the brain with diffusion-weighted imaging and fluid-attenuated inversion recovery (DWI-FLAIR) highlighted a 3.1 x 3.1 x 2.5 centimeter (cm) mixed signal intensity focus most consistent with a pituitary mass, likely reflecting a macroadenoma with potential products of hemorrhage (Figure 2). 

The patient was started on stress-dose hydrocortisone and underwent emergent transsphenoidal resection for PA with no intraoperative complications. Pathology of the sellar mass showed areas of hemorrhage and necrosis confirming PA. Postoperative laboratory investigation highlighted an expected cortisol elevation in the setting of exogenous glucocorticoid administration and otherwise revealed the removed adenoma was non-functioning, as shown in Table 1.

**Table 1:** Endocrinologic laboratory investigation immediately prior to transsphenoidal resection. ACTH: adrenocorticotropic hormone; LH: luteinizing hormone, FSH: follicle stimulating hormone; TSH: thyroid stimulating hormone; T3: triiodothyronine; T4: thyroxine

Laboratory investigations	Results	Reference range
ACTH	9.6 mg/dL	7.2-63.3 mg/dL
Calcium	9.2 mg/dL	8.5-10.5 mg/dL
Cortisol	239.2 mcg/dL	2.5 -17.0 mcg/dL
FSH	0.5 mg/dL	<0.5-17.0 ng/dL
Growth hormone	0.90 mg/dL	0.03-2.47 mg/dL
Insulin growth factor	216 ng/mL	65-222 ng/mL
LH	<0.1 milliUnit/mL	1.0-8.0 milliUnit/mL
Parathyroid hormone	59 pg/mL	12-72 pg/mL
Prolactin	0.6 ng/mL	<0.5-17.0 ng/mL
TSH	1.086 mcUnit/mL	0.40-5.000 mcUnit/mL
T3 total	3.7 pg/mL	1.7-3.7 pg/mL
T4 free	1.14 ng/dL	0.80-1.80 ng/dL

**Figure 3:**
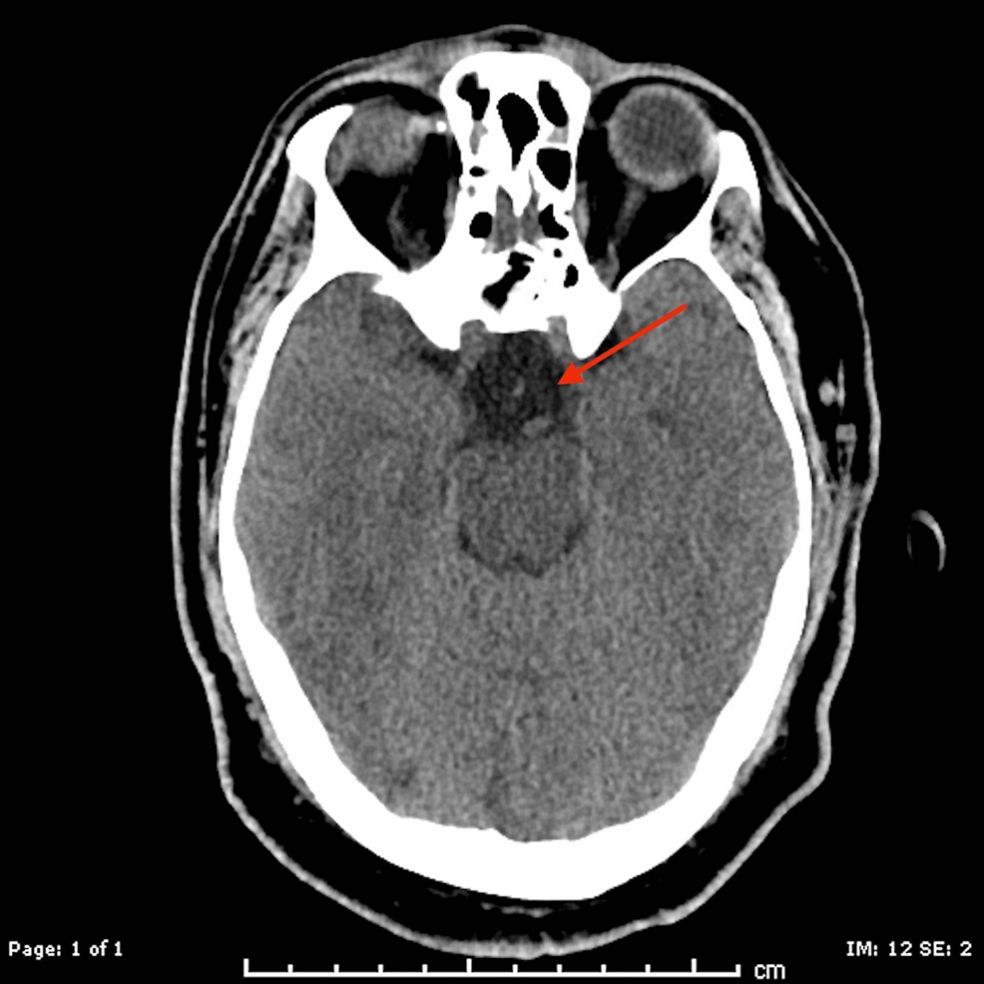
CT head was taken after transsphenoidal resection of the pituitary mass. The arrow shows the interval removal of pituitary mass with no unexpected post-operative changes. CT: computed tomography

CT head obtained after transsphenoidal resection showed no unexpected findings or evidence of hemorrhage or other acute abnormalities (Figure 3).

After the procedure, the patient suffered complications from triphasic antidiuretic hormone (ADH) secretion. This first phase began with hypernatremia and polyuria related to antidiuretic deficiency, for which he was given one dose of intravenous desmopressin at 1 mcg. His sodium corrected quickly; however, this was short-lived as he entered a new hyponatremic phase, clinically consistent with a syndrome of inappropriate ADH secretion (SIADH). The patient was then fluid restricted to 1000 ml daily with an improvement in sodium. Polyuria started once again in the final state of triphasic ADH secretion. He was restarted on oral desmopressin as he could tolerate medications by mouth. The desmopressin dose was decreased throughout admission until discontinued following normalization of the patient’s urine output. During the same time frame, he was also switched from intravenous hydrocortisone to oral hydrocortisone, given hemodynamic stability throughout his hospitalization. It was decided to continue tapering the hydrocortisone as an outpatient until the patient could achieve adequate maintenance dosing based on future cortisol level testing. His post-procedure laboratory values, including free T4 and gonadal hormone testing, were within normal limits, prompting the restart of his home dose of levothyroxine at 25 mcg daily and his previous testosterone supplementation. The patient had complete resolution of diplopia, headache, and nausea. He was discharged on hydrocortisone, levothyroxine, and testosterone, with a closely scheduled follow-up visit in the endocrinology clinic.

## Discussion

The clinical presentation of PA varies and can include severe headache, nausea, vomiting, decreased visual acuity, visual field defects, diplopia, and altered consciousness. Our patient had several classical findings, most notably oculomotor nerve palsy manifesting as right eye ptosis and fixed gaze, which prompted neuroimaging. These visual findings have been described previously as a consequence of oculomotor nerve compression as the pituitary enlarges and impinges upon the cavernous sinus [5]. While our patient was male and in his fifth decade of life, both factors associated with PA in prior case series, he also had a history of hypothyroidism and hypogonadism [6]. Documentation reveals that the patient had primary hypothyroidism and primary hypogonadism, although there is a lack of pre-hospitalization laboratory testing available to confirm this. In regards to hypothyroidism, the role of pituitary imaging is only commonly indicated in the investigation of suspected central hypothyroidism, while with patients suffering from hypogonadism, imaging to rule out lesions in the hypothalamic-pituitary region is recommended for total testosterone concentrations less than 150 ng/dl [7]. In the case of our patient, no formal structural evaluation of his pituitary gland was ever done, which is typical as pituitary macroadenoma is undiagnosed at the time of apoplexy in the majority of cases. Although these conditions indicate an element of pituitary insufficiency, there is limited research on hypogonadism or hypothyroidism as it relates to being a predictive factor for eventual pituitary apoplexy.

Surgical procedures with significant hemodynamic shifts or intraoperative complications and states of systemic illness like sepsis or myocardial infarction have been thought to precipitate apoplexy through excessive pituitary gland stimulation, as the pituitary aims to increase glucocorticoid production in response to stress [8]. In addition, the intrinsic vascularity of a pituitary adenoma is reduced compared with the normal pituitary gland and appears to be more fragile and immature, which, along with the increased metabolic requirement needed for tumor development, may lead to the outgrowth of blood supply [9,10]. This is of particular concern with an acute reduction in systemic blood pressure (hemodynamic shift) that may decrease blood flow to the pituitary adenoma and further precipitate apoplexy [11]. However, only a handful of case reports document postoperative apoplexy in elective surgeries such as hip or knee arthroplasty without hemodynamic compromise, as seen in our patient [12]. Importantly, our patient was receiving prophylactic heparin during his hospital course. While anticoagulation is an established risk factor for PA, it is typically described in the setting of therapeutic anticoagulation. One case report does identify low molecular weight heparin thromboprophylaxis following shoulder arthroplasty as a possible contributory factor in symptomatic pituitary apoplexy, given the lack of other traditional risk factors present in that case. Altogether, though, there remains a lack of evidence for causation regarding venous thromboprophylaxis and PA development, and administration should be based on clinical judgment [13].

Neuroimaging was pursued in our patient to evaluate for stroke, as PA was not initially considered, again likely due to the lack of hemodynamic compromise or intraoperative complications during his procedure. Imaging with CT or MRI usually reveals signs of intra-pituitary or intra-adenoma hemorrhage, stalk deviation, and compression of normal pituitary tissue [14]. Our patients' imaging was consistent with compression of normal pituitary architecture, with products of hemorrhage and necrosis confirmed on biopsy.

Emergent transsphenoidal surgical decompression is indicated in cases with progressive impairment of visual acuity or visual field deficits, as seen in our patient, prompting emergent surgery. In contrast, fully alert patients without visual deficits may be observed. While ophthalmoplegia will typically recover regardless of surgery or conservative management, postoperative recovery of visual function correlates inversely with the time elapsed from the clinical presentation [15].

While visual deficits typically remit with prompt surgery, as evidenced by our patient, pituitary function usually does not recover after the resolution of pituitary hemorrhage, making endocrine hormone replacement with adrenal, thyroid, and gonadotropin analogs necessary. High-dose glucocorticoids should also be used depending on clinical or hemodynamic status, as stress-dose glucocorticoid replacement is crucial if there is acute adrenal insufficiency in the setting of corticotropin deficiency and was started in our patient prophylactically once neuroimaging was obtained. The current standard of care practice for hydrocortisone dosing after transsphenoidal resection is a replacement dosing of 15 to 25 milligrams per day on discharge, and so our patient was tapered to this range. Exogenous corticosteroid dosing is to remain for four to six weeks until a definitive determination of adrenal status can be made with early morning cortisol testing as an outpatient [16].

Notably, he also developed postoperative ADH deficiency, a relatively common complication of transsphenoidal or transcranial surgery, with 50-60% of patients developing ADH deficiency postoperatively [17]. Our patient remained hemodynamically appropriate and was discharged on a hydrocortisone taper in addition to thyroid and gonadal hormone replacement. No dose adjustment was made for thyroid and gonadal hormone replacement therapy in the case of our patient, as subsequent postoperative testing was within normal limits. Following pituitary apoplexy, patients often suffer an acquired form of panhypopituitarism requiring therapy regardless of pre-existing endocrine disorders. There is currently a lack of data regarding the severity of hormone deficiency or length of replacement therapy between patients with and without prior endocrine disorders following transsphenoidal resection. Although it is estimated that approximately 80% of all these patients will require some form of long-term hormone therapy, the most common replacements involve corticosteroids (40-85%), thyroid hormone (50-70%), and gonadal hormones (40-80%) [18].

## Conclusions

Elective orthopedic surgery is a rare cause of apoplexy of pituitary macroadenoma that has not been well described. Our patient, who underwent a right total knee arthroplasty, developed pituitary apoplexy in the postoperative setting and received an emergent transsphenoidal resection after recognition of symptoms on a physical exam. This case report illustrates that even in elective, uncomplicated surgical procedures, sudden-onset neurological and visual symptoms should prompt clinicians to consider pituitary apoplexy, especially in patients with pre-existing endocrine hormone deficiencies.

## References

[1] Doglietto F, Costi E, Villaret AB, Mardighian D, Fontanella MM, Giustina A (2016). New oral anticoagulants and pituitary apoplexy. Pituitary.

[2] Semple PL, Jane JA Jr, Laws ER Jr (2007). Clinical relevance of precipitating factors in pituitary apoplexy. Neurosurgery.

[3] Cinar N, Tekinel Y, Dagdelen S, Oruckaptan H, Soylemezoglu F, Erbas T (2013). Cavernous sinus invasion might be a risk factor for apoplexy. Pituitary.

[4] Möller-Goede DL, Brändle M, Landau K, Bernays RL, Schmid C (2011). Pituitary apoplexy: re-evaluation of risk factors for bleeding into pituitary adenomas and impact on outcome. Eur J Endocrinol.

[5] Cho WJ, Joo SP, Kim TS, Seo BR (2009). Pituitary apoplexy presenting as isolated third cranial nerve palsy with ptosis : two case reports. J Korean Neurosurg Soc.

[6] Dubuisson AS, Beckers A, Stevenaert A (2007). Classical pituitary tumour apoplexy: clinical features, management and outcomes in a series of 24 patients. Clin Neurol Neurosurg.

[7] Dandona P, Rosenberg MT (2010). A practical guide to male hypogonadism in the primary care setting. Int J Clin Pract.

[8] Biousse V, Newman NJ, Oyesiku NM (2001). Precipitating factors in pituitary apoplexy. J Neurol Neurosurg Psychiatry.

[9] Turner HE, Nagy Z, Gatter KC, Esiri MM, Harris AL, Wass JA (2000). Angiogenesis in pituitary adenomas and the normal pituitary gland. J Clin Endocrinol Metab.

[10] Hirano A, Tomiyasu U, Zimmerman HM (1972). The fine structure of blood vessels in chromophobe adenoma. Acta Neuropathol.

[11] Briet C, Salenave S, Bonneville JF, Laws ER, Chanson P (2015). Pituitary apoplexy. Endocr Rev.

[12] Madhusudhan S, Madhusudhan TR, Haslett RS, Sinha A (2011). Pituitary apoplexy following shoulder arthroplasty: a case report. J Med Case Rep.

[13] Goel V, Debnath UK, Singh J, Brydon HL (2009). Pituitary apoplexy after joint arthroplasty. J Arthroplasty.

[14] Elsässer Imboden PN, De Tribolet N, Lobrinus A, Gaillard RC, Portmann L, Pralong F, Gomez F (2005). Apoplexy in pituitary macroadenoma: eight patients presenting in 12 months. Medicine (Baltimore).

[15] Ayuk J, McGregor EJ, Mitchell RD, Gittoes NJ (2004). Acute management of pituitary apoplexy--surgery or conservative management?. Clin Endocrinol (Oxf).

[16] Inder WJ, Hunt PJ (2002). Glucocorticoid replacement in pituitary surgery: guidelines for perioperative assessment and management. J Clin Endocrinol Metab.

[17] Fatemi N, Dusick JR, Mattozo C (2008). Pituitary hormonal loss and recovery after transsphenoidal adenoma removal. Neurosurgery.

[18] Ranabir S, Baruah MP (2011). Pituitary apoplexy. Indian J Endocrinol Metab.

